# Saints on Earth

**Published:** 1887-05-14

**Authors:** 


### SAINTS ON EARTH.

The Saints above are stars in heaven—
What are the Saints on earth?
Like trees they stand whom God has given,
Our Eden's happy birth.
Faith is their fixed, unswerving root,
Hope their unfading flower,
Fair deeds of charity their fruit,
The glory of their bower.

KEBLE.

